# Effects of JSOG-6 on protection against bone loss in ovariectomized mice through regulation of osteoblast differentiation and osteoclast formation

**DOI:** 10.1186/1472-6882-14-184

**Published:** 2014-06-06

**Authors:** Hwa-Jin Chung, Lan Cho, Joon-Shik Shin, Jinho Lee, In-Hyuk Ha, Hyen Joo Park, Sang Kook Lee

**Affiliations:** 1 College of Pharmacy, Natural Products Research Institute, Seoul National University, San 56-1 Sillim-dong, Gwanak-gu, Seoul 151-742, Korea; 2 Jaseng Spine and Joint Research Institute, Jaseng Medical Foundation, Jaseng Hospital of Korean Medicine, Seoul 135-896, Korea

**Keywords:** JSOG-6, Ovariectomized mice, Bone loss, Osteoclast, Osteoblast

## Abstract

**Background:**

JSOG-6 is used as a traditional medicine to relieve the symptoms associated with inflammation, rheumatism, and osteoporosis in Korea. In the present study, we investigated the effects of JSOG-6 on bone loss prevention both in *in vitro* and *in vivo* as well as its underlying mechanism of action.

**Methods:**

Protection against bone loss was assessed in an ovariectomized (OVX) mouse model. Bone microarchitecture was measured using a micro-computed tomography to detect the parameters of three-dimensional structure of a trabecular bone. Serum biomarkers were also evaluated in an OVX-induced model. Osteoclasts derived from mouse bone marrow cells (BMCs) and osteoblastic MC3T3-E1 cells were also employed to investigate the mechanism of action.

**Results:**

Oral administration of JSOG-6 significantly increased the bone mineral density (BMD) of the femur in OVX mice *in vivo*. Especially, the reduced Tb.No (trabecular bone number) in the OVX group was significantly recovered by JSOG-6 treatment. The serum levels of alkaline phosphatase (ALP), osteocalcin, C-terminal telopeptide, and tartrate-resistant acid phosphatase, biomarkers of bone resorption, were significantly elevated in OVX mice, but JSOG-6 effectively inhibited the increase in OVX mice. JSOG-6 was also found to enhance the osteoblastic differentiation and maturation with the increase of the density and ALP activity, a marker of osteoblastic differentiation, as well as calcium deposition, a marker of osteoblastic maturation in MC3T3-E1 cells. The effects of JSOG-6 on osteoblastic differentiation were also associated in part with the increase of ALP and OPN mRNA expressions and the decrease of RANKL mRNA expression in MC3T3-E1 cells.

**Conclusions:**

The findings demonstrate that JSOG-6 induced protection against bone loss in OVX mice, and its anti-osteoporotic property might be, in part, a function of the stimulation of osteoblast differentiation and the inhibition of osteoclast formation. These findings suggest that JSOG-6 might be an applicable therapeutic traditional medicine for the regulation of the osteoporotic response.

## Background

Osteoporosis is an age-dependent metabolic bone disease characterized by the decrease in bone mass, the deterioration of bone tissue, and an increased risk of fractures [1,2]. In the process of osteoporosis, the bone mineral density (BMD) is decreased, bone microarchitecture is deteriorated, and a variety of proteins in bone are also altered. Bone mass is regulated by continuous bone remodeling through bone resorption and bone formation by osteoclasts and osteoblasts, respectively [3,4]. Indeed, bone homeostasis depends on maintaining a balance between the activities of bone-forming osteoblasts and bone-resorbing osteoclasts [5,6], which is ultimately determined by the proliferation and differentiation of progenitors of these two bone-associated cells.

Osteoclasts are derived from hematopoietic cells within the monocyte/macrophage lineage [7] and regulated by the combined action of the receptor activator of the NF-κB ligand (RANKL) [8]. However, osteoblasts induce the increase in bone mass by inhibiting the ability of osteoclasts. Osteoblasts also express RANKL and osteoprotegerin (OPG), a decoy receptor of RANKL, which are essential factors regulating the formation and activation of osteoclasts (osteoclastogenesis) [9,10]. Therefore, the balance of RANKL and OPG mainly contributes to bone remodeling because RANKL stimulates osteoclastogenesis, while OPG suppresses bone resorption [11]. Yet, excessive osteoclastogenesis and defects in osteoblastogenesis are associated with bone diseases such as osteoporosis and rheumatoid arthritis [12].

JSOG-6 (named Bogangyeongol-hwan) consists of a mixture of six crude drugs, and each drug has been widely used in traditional medicine to treat various bone disorders such as arthritis, degenerative disc disease and osteoporosis. Of the six components of JSOG-6, *Harpagophytum procumbens* var. *sublobatum* (Engl.) Stapf, radix, called Devil’s claw, is an herbal substance commonly used by patients with osteoarthritis (OA). Anti-inflammatory activities of the root extracts of *H. procumbens* var. *sublobatum* have also been reported in various inflammation models [13-15]. Another component of JSOG-6, *Drynaria fortunei* (Kunze ex Mett.) J. Sm., rhizome also showed an anti-osteogenic effect in a bone-resorption model [16] as well as anti-inflammatory properties [17]. *Poria cocos* F.A.Wolf, sclerotium [18] and *Rehmannia glutinosa* (Gaertn.) DC*.*, radix [19] exhibited anti-inflammatory effects. Furthermore, *Panax ginseng* C.A.Mey*.,* radix showed an anti-osteoporotic effect in an OVX rat model [20]. These reported data suggest that JSOG-6 might have the potential to alleviate the symptoms of bone loss-associated diseases.

In the present study, we investigated the activities of JSOG-6 *in vitro* and in *in vivo* bone-remodeling models and examined its underlying molecular mechanism.

## Methods

### Preparation of test samples

A mixture of six crude drugs (*Harpagophytum procumbens* var. *sublobatum* (Engl.) Stapf, radix (120 g), *Drynaria fortunei* (Kunze ex Mett.) J. Sm., rhizome (120 g), *Equus asinus* L., gelatinized (120 g), *Poria cocos* F.A.Wolf, sclerotium (120 g), *Rehmannia glutinosa* (Gaertn.) DC*.*, radix (120 g), and *Panax ginseng* C.A.Mey*.,* radix (120 g)) was boiled in tap water (10 L) for 6 h, and the extract was freeze-dried to obtain the JSOG-6 extracts (259 g, 35.6%). The crude drugs were purchased from an herbal market in Seoul, Korea, and authenticated by Dr. S.H. Lee, Jaseng Hospital of Korean Medicine in Seoul, Korea. Voucher specimens of the plants used in this study were deposited in the herbarium at Jaseng Hospital of Oriental Medicine.

### Animals

Female ICR mice (18–20 g, ~8 weeks old) were purchased from Central Laboratory Animal Inc. (Seoul, Korea). Animals were housed under standard laboratory conditions with free access to food and water. The temperature was thermostatically regulated to 22°C ± 2°C, and a 12-hour light/dark schedule was maintained. Prior to their use, they were allowed one week for acclimatization within the work area environment. All animal experiments were carried out in accordance with the Institutional Animal Care and Use Committee Guidelines of Ewha Womans University (permission number: EWHA2010-2-07).

At 9 weeks of age, mice were bilaterally OVX, and 8 mice were Sham-operated (Sham). After 1 week of recovery from surgery, the OVX mice were randomly divided into 5 groups of 8 mice per group (OVX control, 17β-estradiol (E2, 20 μg/kg), and JSOG-6 (50, 150, or 450 mg/kg)). JSOG-6 was orally administered in distilled water (0.3 mL) for 12 weeks, and the same volume of distilled water was used in the Sham- and OVX control groups. After 12 weeks of treatment, the animals were sacrificed, and blood samples were collected for serum isolation. The femur bones were dissected and divested of soft tissue for analysis of trabecular microarchitecture.

### Analysis of serum bone biomarkers

Serum calcium (Ca) and phosphorus (P) were measured as previously described [21]. The serum concentrations of osteocalcin (OCN) and alkaline phosphatase (ALP) activity were assayed using an ELISA kit (Biomedical Technologies Inc., Stoughton, MA, USA) and QuantiChrome ALP assay kit (DALP-250, BioAssay Systems, CA, USA) according to the manufacturer’s instructions, respectively. The serum levels of C-terminal telopeptides (CTx), bone resorption biomarkers that indicate osteoclastic activity, were also analyzed using commercial ELISA kits (Serum CrossLaps, Nordic Bioscience, Herlev, Denmark). The tartrate-resistant acid phosphatase (TRAP) concentration was determined by a mouse TRAP assay kit (Suomen Bioanalytikka Oy, Turku, Finland).

### Analysis of bone microarchitecture

Bone microarchitecture of the femur was scanned using a micro-computed tomography (μCT system, SkyScan 1076, Aart-selaar, Belgium) in the region of 0.6–2.1 mm from the growth plate. The X-ray source was set at a voltage of 50 kV and a current of 200 μA and filtered with a 0.5 mm aluminum filter. The scanning angular rotation was 180° with an angular step of 0.5°. The voxel size was fixed at 8.9 μm. The morphometric index of the bone region was determined from the microtomographic data using a 3D image (SkyScan). The following measures characterizing the three-dimensional structure of a trabecular bone were determined: the ratio of bone components to volume of interest (BV/TV,%), trabecular thickness (Tb.Th, mm), trabecular separation (Tb.Sp, mm), trabecular bone number (Tb.N, mm-1), and structure model index (SMI).

BV/TV indicates the ratio which a trabecular bone is occupied with a given volume of interest, usually measured as a% value. Tb.Th and Tb.Sp define the shape of a trabecular bone, whereas Tb.N implies the number of traversals made per unit length on a random linear path across a trabecular bone through a given volume of interest. SMI quantifies the relative prevalence of rod-, plate-, or sphere-shapes in a trabecular bone structure.

### Chemicals

α-Modified minimal essential medium (α-MEM), fetal bovine serum (FBS), sodium pyruvate, L-glutamine, antibiotic-antimycotic solution, and trypsin-EDTA were purchased from Invitrogen Co. (Grand Island, NY, USA). RANKL and macrophage-colony stimulating factor (M-CSF) were purchased from R&D systems (Minneapolis, MN, USA). Ascorbic acid, β-glycerophosphate,3-(4,5-dimethylthiazol-2-yl)-2,5-diphenyltetrazolium bromide (MTT), and other chemicals were obtained from Sigma (St. Louis, MO, USA) unless otherwise indicated.

### Cell culture

Mouse calvaria MC3T3-E1 cells, obtained from American Type Culture Collection (ATCC, Rockville, MD, USA), were cultured in α-MEM supplemented with 10% heat-inactivated FBS, 100 units/mL penicillin, 100 μg/mL streptomycin, and 0.25 μg/mL amphotericin B. Cells were incubated at 37°C and 5% CO_2_ in a humidified atmosphere.

### Evaluation of growth inhibitory potential

MC3T3-E1 cells (1 × 10^4^ cells/mL in 96-well plates) were treated with various concentrations of JSOG-6 and incubated at 37°C in a humidified atmosphere with 5% CO_2_. After JSOG-6 treatment for 72 h, MTT solution (5 mg/mL in PBS) was added to the medium (final concentration 500 μg/mL) and further incubated for 4 h. The medium was discarded and 200 μL of (100% or 10%) DMSO was added to each well to dissolve the formazan. The absorbance was measured at 570 nm. The effect of JSOG-6 on cell growth was calculated as a percentage relative to solvent-treated control incubations, and the IC_50_ values were calculated using non-linear regression analysis (percent cell proliferation versus concentration).

### Alkaline phosphatase (ALP) activity

The ALP activity was determined according to a previously described method [22]. MC3T3-E1 cells were incubated in osteogenic medium containing 400 μM ascorbic acid and 5 mM β-glycerophosphate. The cells (2 × 10^4^ cells/mL) were incubated with or without various concentrations of JSOG-6. After 4 days, the cells were washed with PBS and fixed with 70% ethanol for 5 min and then extracted into lysis solution (10 mM Tris, 0.1% Triton X-100 buffer (pH 7.5). Enzyme activity was determined using *p*-nitrophenylphosphate (*p*-NPP) as a substrate. The color change reflecting the conversion of *p*-NPP to *p*-nitrophenol was measured at 405 nm. The protein concentration of cell lysates was measured using the Bradford assay.

### Mineralization assay

The calcium deposition of MC3T3-E1 cells was determined by a previously reported method [23]. MC3T3-E1 cells (2 × 10^4^ cells/mL) were incubated with or without various concentrations of JSOG-6 for 14 days. The cells were washed twice with PBS and fixed with 70% ethanol for 30 min. The fixed MC3T3-E1 cells were stained with 2% Alizarin Red S solution (pH 4.0) for 5 min. The plate was washed several times by distilled water, and then the cells were observed under a microscope.

### Real-time reverse transcriptase-polymerase chain reaction (Real-time RT-PCR)

Lipopolysaccharide (LPS), a cell component of Gram-negative bacteria, is an important mediator of pathological bone destruction associated with inflammation. MC3T3-E1 cells were stimulated with 1 μg/mL LPS in the presence or absence of JSOG-6 for 48 h. Total cellular RNA was extracted with TRI reagent (Sigma, St. Louis, MO, USA) according to the manufacturer’s recommended procedure. Total RNA (1 μg) was reverse-transcribed using oligo-(dT)_15_ primers and avian myeloblastosis virus (AMV) reverse transcriptase (Promega, Madison, WI, USA).

Real-time RT-PCR was conducted with a MiniOpticon system (Bio-Rad, Hercules, CA, USA) using 5 μL of the reverse-transcription product, iQ™ SYBR® Green Supermix (Bio-Rad, Hercules, CA, USA) and primers for a total volume of 20 μL. Standard thermal cycler conditions were employed (95°C for 20 s, 40 cycles of 95°C for 20 s, 56°C for 20 s, and 72°C for 30 s, followed by 95°C for 1 min, and 55°C for 1 min). The threshold cycle (C_T_), the fractional cycle number at which the amount of amplified target gene reaches a fixed threshold, was determined by MJ Opticon Monitor software. The mean threshold cycle (C_T_) value for each transcript was normalized by dividing it by the mean C_T_ value for the β-actin transcript for that sample. Normalized transcript levels were expressed relative to those obtained from the control. Real-time RT-PCR primer sequences are listed in Table 1.

**Table 1:** Sequences of target gene-specific primers used in real-time PCR

**Target genes**		** Sequences**	**Accession number**
ALP	Sense	5′-ATGCCCTGAAACTCCAAA-3′	NM_001287176
	Antisense	5′-AGACGCCCATACCATCTC-3′	
OPN	Sense	5′-GCTTGGCTTATGGACTGA-3′	NM_001204201
	Antisense	5′-GGCAACAGGGATGACATC-3′	
OCN	Sense	5′-CAGACAAGTCCCACACAG-3′	NM_007541
	Antisense	5′-GCAGAGTGAGCAGAAAGA-3′	
OPG	Sense	5′-TGGGAGAAGAACCTTATTTTG-3′	NM_008764
	Antisense	5′-CCAGCATCCTCTTTCATAAAG-3′	
RANKL	Sense	5′-CCATGAAAACGCAGATTTG-3′	NM_011613
	Antisense	5′-CCCTGAAAGGCTTGTTTC-3′	
β-actin	Sense	5′-AAGGCCAACCGTGAAAAGAT-3′	NM_007393
	Antisense	5′-GTGGTACGACCAGAGGCATAC-3′	

### Preparation of total cell lysates

MC3T3-E1 cells (5 × 10^5^ cells/mL in 60 mm dish) were incubated with or without various concentrations of JSOG-6 for 48 h. To obtain total cell lysates, the cells were washed with ice-cold PBS and lysed in boiling 2X sample loading buffer (250 mM Tris–HCl (pH 6.8), 4% SDS, 10% glycerol, 0.006% bromophenol blue, 50 mM sodium fluoride, 5 mM sodium orthovanadate, and 2% β-mercaptoethanol). Cell lysates were boiled for an additional 20 min and stored at −20°C. The protein content of cell lysates was determined by the BCA method.

### Western blot analysis

Equal amounts of cell lysates (40 ~ 50 μg) were subjected to 8% and 10% SDS-PAGE and electrotransferred onto polyvinylidene difluoride (PVDF) membranes (Millipore, MA, USA). Membranes were blocked in PBST (PBS with 0.1% Tween-20) containing 5% non-fat dry milk for 1 h at room temperature. After washing 3 times with PBST, membranes were incubated with primary antibodies against OPG, RANKL, c-Fos, and TRAF6 (Santa Cruz Biotechnology, Santa Cruz, CA, USA), p-ERK, ERK (Cell Signaling, Danvers, MA, USA), and β-actin (Sigma) for 3 h at room temperature or overnight at 4°C. Membranes were washed 3 times with PBST and incubated with the corresponding secondary antibodies (Santa Cruz) for 90 min at room temperature. The blots were washed 3 times with PBST and visualized using an enhanced chemiluminescence (ECL) Western blotting detection system (Lab Frontier, Suwon, Korea).

### Murine bone marrow-derived osteoclasts

BMCs were isolated from 4-week-old mice as previously described [24]. The cells were plated into 96-well plates in 30 ng/mL of M-CSF for 24 h. Next, the cells were treated with the indicated concentrations of JSOG-6 in the presence of 100 ng/mL of RANKL and 30 ng/mL of M-CSF. The medium was replaced with 50% volume with and without JSOG-6 every 2 days, and the culture was terminated after 5 days.

### Tartrate-resistant acid phosphatase (TRAP) staining

Osteoclast differentiation was assessed by TRAP (Sigma-Aldrich, St. Louis, MO, USA) activity. 5 days after stimulating the cells with M-CSF and RANKL (100 and 30 ng/mL, respectively), the cells were washed with PBS and fixed with 4% paraformaldehyde for 5 min. The cells were rinsed in de-ionized water and incubated in tartrate-staining solution in the dark for 1 h at 37°C. The cells were rinsed in de-ionized water and allowed to air dry. TRAP-positive multinucleated cells containing 3 or more nuclei were counted as osteoclasts.

### Statistics

All experiments were repeated at least 3 times. Data are presented as the mean ± SD for the indicated number of independently performed experiments. The statistical significance within a parameter was evaluated by one-way analysis of variation (ANOVA) coupled with Dunnett’s *t*-test.

## Results

### Effect of JSOG-6 on bone loss in an OVX-induced mouse model

**Figure 1:**
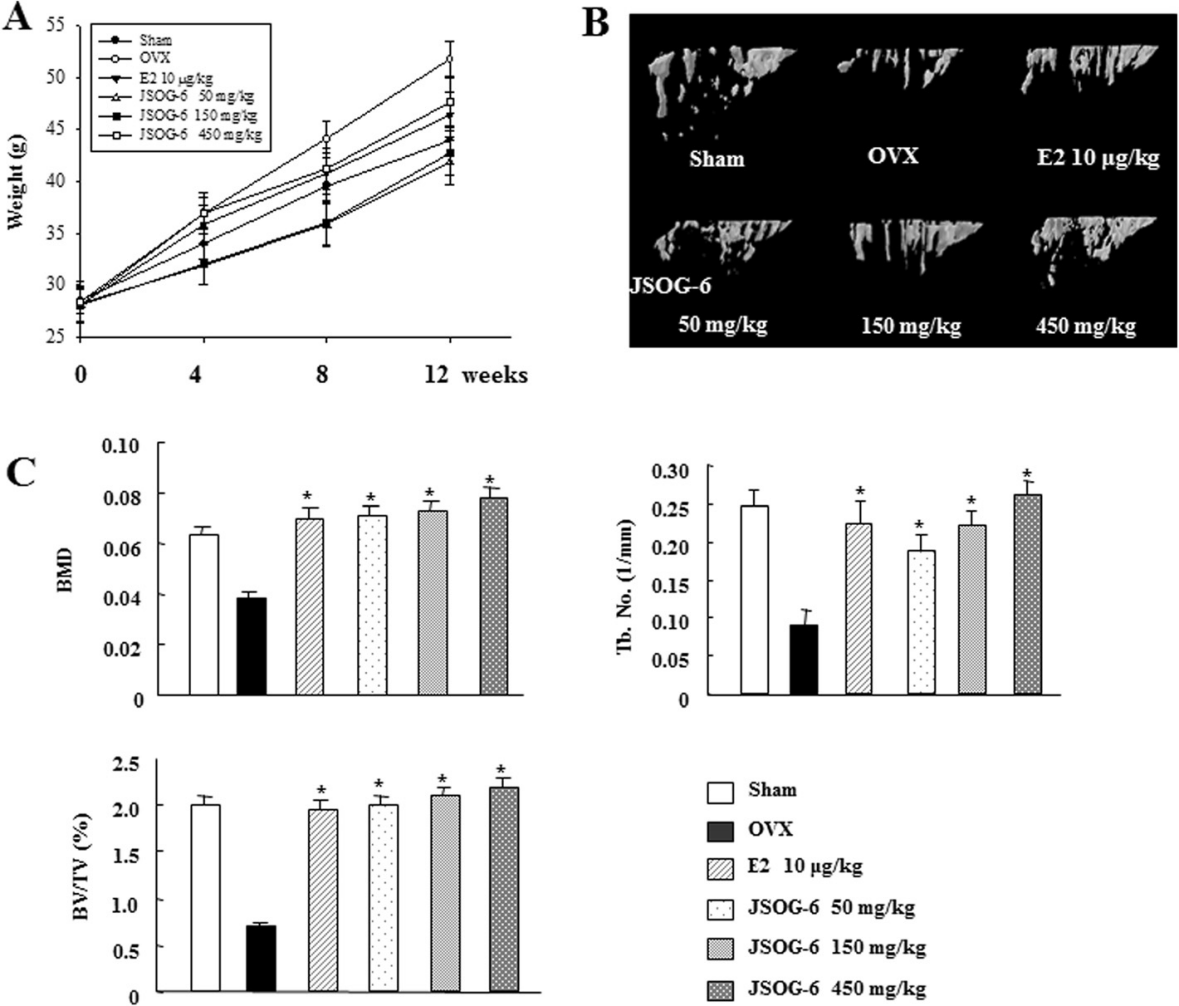
**Effect of JSOG-6 on bone loss in OVX mice. (A)** Change in body weight 12 weeks after ovarietomy. **(B)** Effect of JSOG-6 on bone 3D microCT image of the distal femur in OVX mice. **(C)** Effect of JSOG-6 on the bone morphometric parameters BMD, BV/TV (%), and Tb.No (1/mm) as analyzed with micro-CT SkyScan CTAN software. Data represent the mean ± S.D. (n = 8). *P < 0.01 indicates statistically significant differences from the OVX mice group.

The anti-osteoporotic activity of JSOG-6 was primarily performed in an *in vivo* experiment employing an OVX-induced bone loss mouse model. Body weight change was monitored during the administration of JSOG-6. As shown in Figure 1A, the increase in body weight in the OVX group was significantly higher than that in the Sham-operation group (*P* < 0.01). However, mice given an oral administration of JSOG-6 for 12 weeks after OVX showed a relatively slow increase in body weight compared to the OVX group. The estradiol (E2, 10 μg/kg)-treated group showed a similar result. The destruction of the trabecular bone of the femur was also observed by 3D-μCT. As illustrated in Figure 1B, OVX caused a significant deterioration of trabecular bone architecture compared with the Sham-control group (*P* < 0.01). However, treatment with JSOG-6 retarded or recovered the destruction of the trabecular bone of the femur in a dose-dependent manner in the OVX-induced bone loss model. The protective effect on trabecular bone microarchitecture was also clearly demonstrated by treatment with E2, a positive control, in the same experimental condition. In addition, the microstructural index parameters were observed in the OVX-induced bone loss model (Figure 1C). The BMD of the OVX group was markedly reduced by 38.7% in comparison with the Sham group. The BMD of the trabecular bone of the femur in mice treated with JSOG-6 (50 mg/kg) was shown to be 83.0% higher than that in the OVX group and was not significantly different from that in the Sham control group (*P* < 0.01). E2 also increased the BMD by 80.9% in the OVX-induced bone loss mouse model. The BV/TV was also markedly reduced in the OVX group (64.7%) compared with the Sham group, but the treatment of OVX mice with JSOG-6 or E2 resulted in a significant increase in the BV/TV in the OVX-induced bone loss model (*P* < 0.01). The Tb.No in the OVX group was 63.8% lower than that of the Sham group, but JSOG-6 treatment (50, 150, and 450 mg/kg) significantly recovered the Tb.No value to 113.3, 145.6, and 192.2%, respectively, in a dose-dependent manner than the OVX group. The E2-treated group also showed a 150% recovery of Tb.No compared to the OVX group.

### Effect of JSOG-6 on serum biochemical markers in the OVX mouse model

The effects of JSOG-6 on the bone metabolic biomarkers were also determined with the serum of blood samples collected in OVX mice. The serum levels of calcium and potassium were not significantly different in the Sham, JSOG-6 treatment, and OVX groups (*P* < 0.01) (Table 2). The serum levels of the bone formation markers ALP and OCN were significantly increased in the OVX group compared to the Sham group. However, JSOG-6 treatment decreased the elevated serum levels of ALP and OCN in the OVX mice (*P* < 0.01). In addition, the serum level of TRAP, which is responsible for enhanced osteoclastogenesis and activation of mature osteoclasts for bone resorption, was increased in the OVX group, but JSOG-6 treatment significantly reduced TRAP activity in a dose-dependent manner (*P* < 0.01). The increase in an additional bone resorption marker, CTx, in the OVX group was also inhibited by JSOG-6 treatment (*P* < 0.01) (Table 2).

**Table 2:** Effect of JSOG-6 on the serum parameters in OVX mice

	**Calcium concentration**	**Potassium concentration**	**ALP activity**	**TRAP activity**	**Osteocalcin level**	**CTx level**
Sham	9.1 ± 0.4	8.0 ± 0.3	67.6 ± 2.8	9.1 ± 0.9	148.9 ± 5.3	69.5 ± 4.3
OVX	9.3 ± 0.3	8.3 ± 0.4	85.0 ± 3.1	19.8 ± 1.0	176.9 ± 4.7	95.7 ± 3.8
E2	9.1 ± 0.6	7.9 ± 0.2	59.9 ± 3.1*	10.8 ± 1.0*	141.9 ± 4.9*	76.6 ± 4.7*
JSOG-6						
50 mg/kg	9.4 ± 0.5	7.9 ± 0.3	59.3 ± 3.8*	11.6 ± 1.4*	165.0 ± 6.6	92.3 ± 3.6
150 mg/kg	9.4 ± 0.6	7.9 ± 0.3	62.8 ± 4.6*	9.7 ± 1.1*	155.8 ± 5.1*	89.7 ± 5.2
450 mg/kg	9.6 ± 0.4	8.1 ± 0.4	65.9 ± 3.4*	8.3 ± 0.7*	153.0 ± 5.3*	83.6 ± 3.8*

The serum levels of calcium, potassium, ALP, CTx, OCN, and TARP were analyzed as described in the Methods. Data represent the mean ± S.D. (n = 8). *P < 0.01 indicates statistically significant differences from the OVX mice group.

### Effect of JSOG-6 on the ALP activity of MC3T3-E1 cells

**Figure 2:**
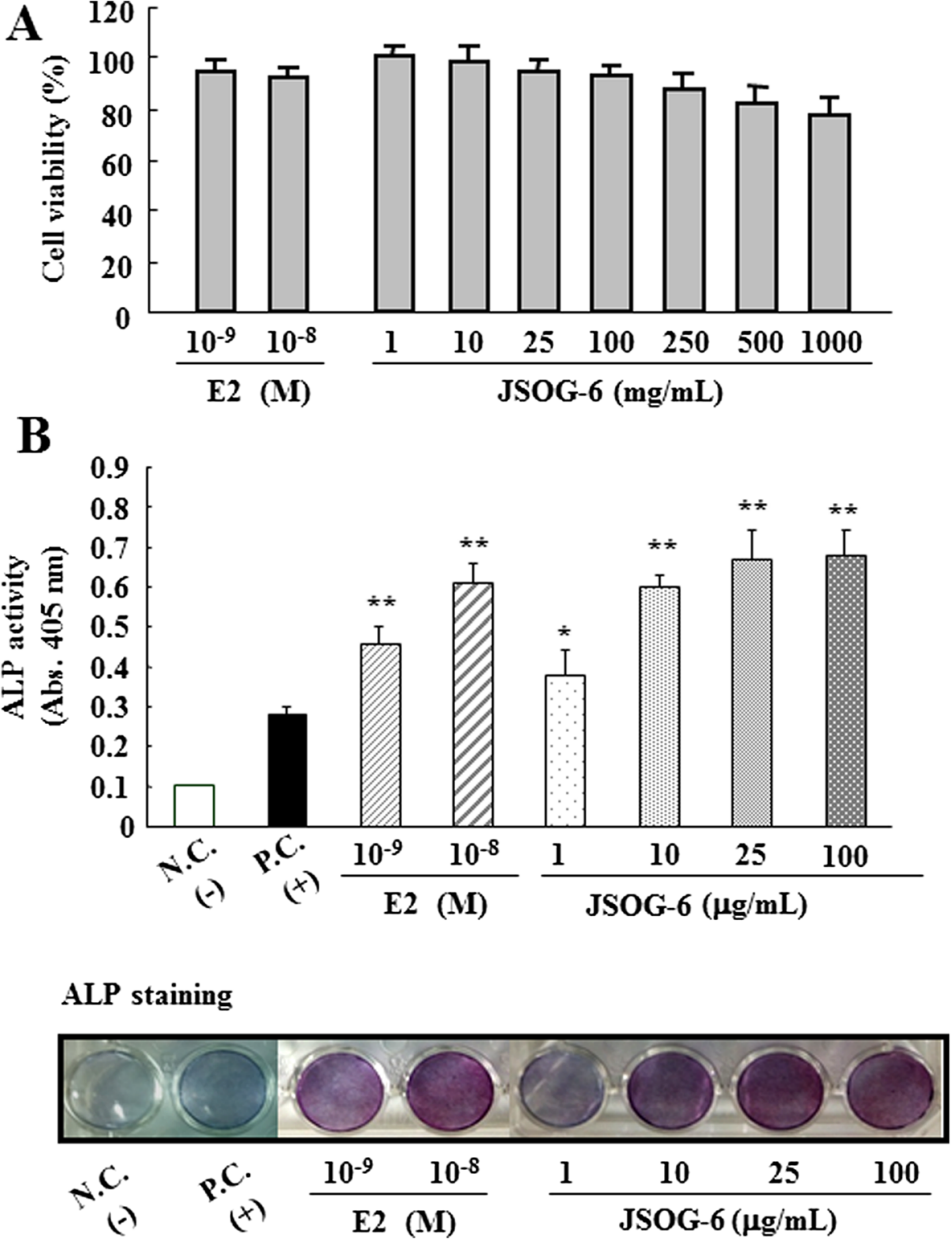
**Effect of JSOG-6 on ALP activity in MC3T3-E1 cells. (A)** Cell viability was measured by the MTT method as described in the Methods. **(B)** MC3T3-E1 cells (2 × 10^4^ cells/mL) were incubated with JSOG-6 in the presence of ascorbic acid and β-glycerophosphate for 4 days. The ALP activity was corrected for the amount of protein. Data represent the mean ± S.D. (n = 3). *P < 0.05, **P < 0.01 indicates statistically significant differences from the control group. N.C., negative control; P.C., positive control (ascorbic acid + β-glycerophosphate).

The ALP staining activity, a marker of osteoblastic differentiation [25,26], was evaluated in osteoblastic MC3T3-El cells. There was no effect on MC3T3-El cell viability with JSOG-6 treatment up to 100 μg/mL as determined by the MTT assay (>95% cell viability) (Figure 2A). Therefore, the cells were treated with up to 100 μg/mL JSOG-6 to understand the biological effects of JSOG-6 without causing cytotoxicity. The MC3T3-El cells were differentiated in the presence of ascorbic acid and β-glycerophosphate in the cell culture medium. After 4 days of differentiation, JSOG-6 was found to enhance the density and ALP activity in a concentration-dependent manner (Figure 2B).

### Effect of JSOG-6 on the mineralization of MC3T3-E1 cells

**Figure 3:**
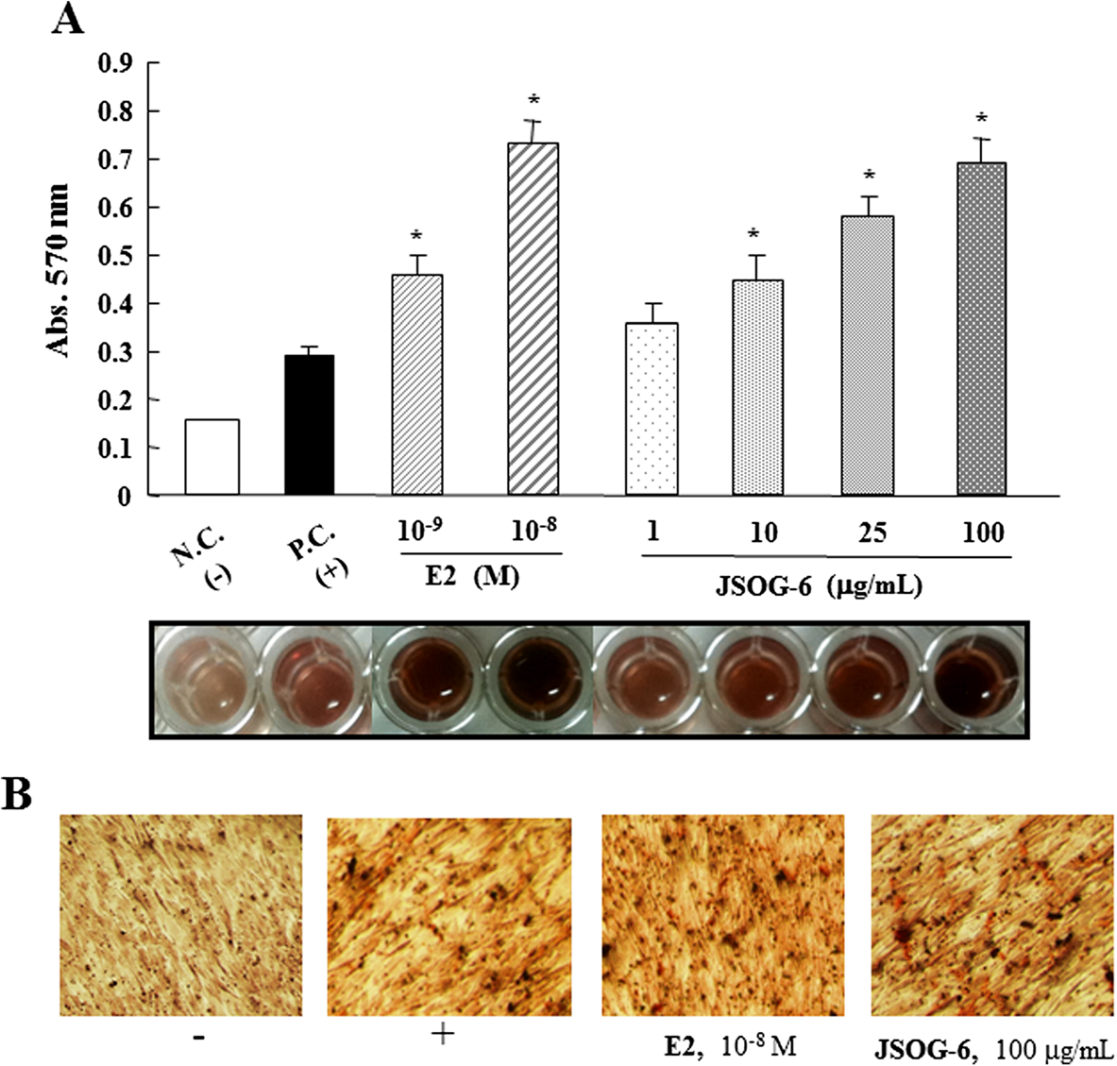
**Effect of JSOG-6 on the mineralization of MC3T3-E1 cells. (A)** MC3T3-E1 cells (2 × 10^4^ cells/mL) were incubated with JSOG-6 in the presence of ascorbic acid and β-glycerophosphate for 14 days. Mineralized nodule formation was assessed by Alizarin red S staining. Data represent the mean ± S.D. (n = 3). *P < 0.01 indicates statistically significant differences from the control group. **(B)** Representative microscopic observation of JSOG-6 on the formation of calcification nodules with staining Alizarin red S. N.C., negative control; P.C., positive control (ascorbic acid + β-glycerophosphate).

Extracellular matrix mineralization is one of the markers of osteoblastic maturation. Alizarins red S staining was used to evaluate extracellular matrix calcium deposition. As illustrated in Figure 3, when the cells were simultaneously treated with JSOG-6 for 14 days, the calcification nodules, represented in red, in MC3T3-E1 cells were increased in a concentration-dependent manner. These results indicate that the effect of JSOG-6 on the ALP activity and calcium deposition stimulated osteoblast differentiation.

### Effect of JSOG-6 on osteoblastic gene expression

**Figure 4:**
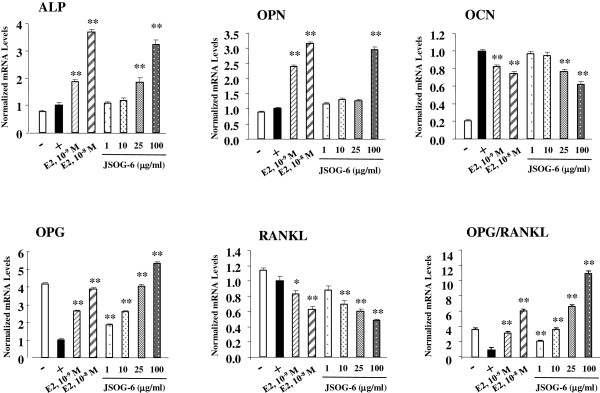
**Effect of JSOG-6 on osteoblastic gene expression.** MC3T3-E1 cells (2 × 10^4^ cells/mL) were treated with the indicated concentrations of JSOG-6 for 48 h, and the mRNA levels of osteoblastic genes were examined using real-time PCR. The results are presented as a relative expression level compared to unstimulated cells and were normalized to β-actin. Data represent the mean ± S.D. (n = 3). *P < 0.05, **P < 0.01 indicates statistically significant differences from the control group.

The effects of JSOG-6 on osteoblast differentiation was further elucidated using the analysis of expression of osteogenic differentiation mediators mRNA by real-time RT-PCR. After 2 days of differentiation, JSOG-6 (100 μg/mL) caused a significant (*P* < 0.01) increase in the expressions of ALP and OPN mRNA, whereas the expression of OCN mRNA was decreased by JSOG-6 treatment. OPG and RANKL play important roles in the maintenance of bone mass and the regulation of bone remodeling. The OPG/RANKL ratio is the major index of osteoclastogenic stimulation. JSOG-6 treatment induced the expression of OPG mRNA in a concentration-dependent manner, whereas JSOG-6 significantly suppressed the expression of RANKL mRNA (*P < 0.05, **P < 0.01). In addition, JSOG-6 significantly increased the OPG/RANKL ratio in MC3T3-E1 cells (*P < 0.01) (Figure 4). These findings suggest that JSOG-6 might modulate the process of osteoclastogenesis via its effect on the OPG/RANKL gene expressions.

### Effect of JSOG-6 on the protein levels of OPG, RANKL, and ERK in MC3T3-E1 cells

**Figure 5:**
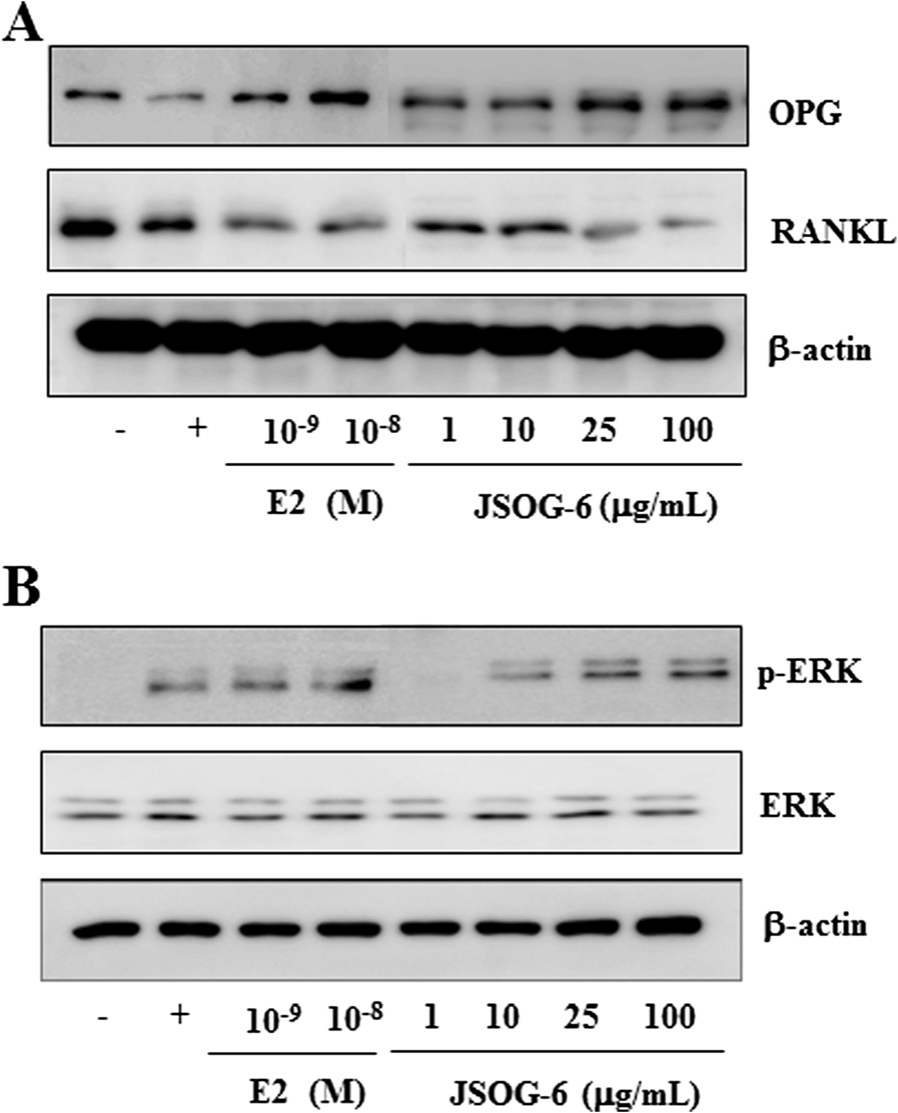
**Effect of JSOG-6 on the protein levels of OPG, RANKL, and ERK in MC3T3-E1 cells. (A, B)** MC3T3-E1 cells (2× 10^4^ cells/mL) were incubated for 48 h and then treated with JSOG-6 for 48 h. After incubation, total cell extracts were obtained and subjected to Western blot analysis as described in the Methods. Data were representative of three separate experiments. β-Actin was used as an internal standard.

The effect of JSOG-6 on the protein expressions of OPG, RANKL, and ERK in MC3T3-E1 cells was evaluated to confirm the activity of JSOG-6 on osteogenic differentiation. As shown in Figure 5A, JSOG-6 increased the protein expression of OPG in a concentration-dependent manner, but the protein expression of RANKL was suppressed. In addition, JSOG-6 treatment induced ERK phosphorylation (Figure 5B).

### Effect of JSOG-6 on osteoclast differentiation

**Figure 6:**
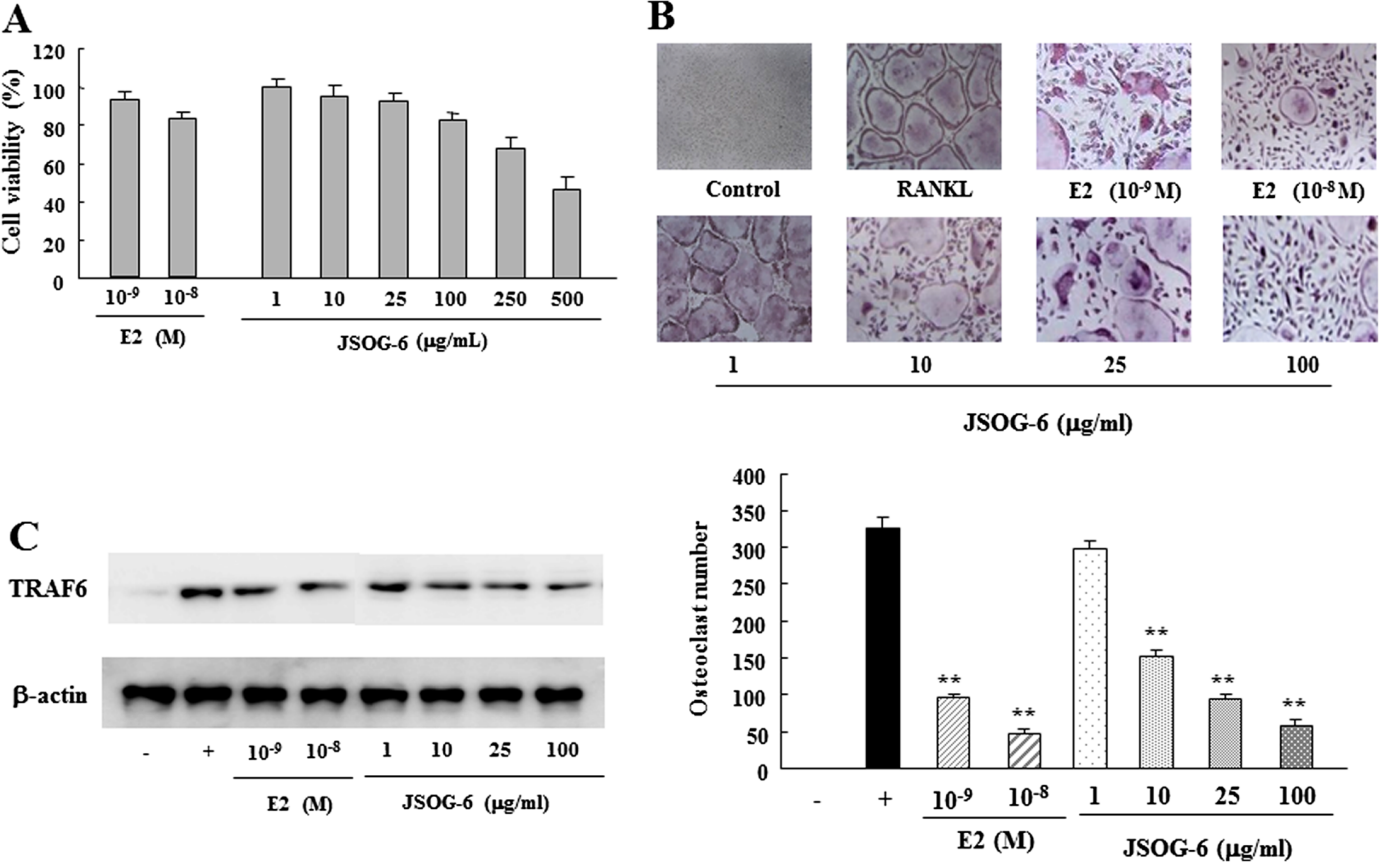
**Effect of JSOG-6 on RANKL-induced osteoclast differentiation. (A)** Cell viability was measured by the MTT method as described in the Methods. **(B)** Bone marrow cells (1 × 10^4^ cells/mL) were incubated with JSOG-6 in the presence of M-CSF (30 ng/mL) and RANKL (100 ng/mL) for 5 days. Osteoclastogenesis was confirmed by TRAP staining. Data represent the mean ± S.D. (n = 3). **P < 0.01 indicates statistically significant differences from the control group. **(C)** The expression of TRAP was determined by Western blot analysis as described in the Methods. Data are representative of three separated experiments. β-Actin was used as an internal standard.

TRAP staining was performed to evaluate the effect of JSOG-6 on RANKL-induced osteoclast differentiation. As depicted in Figure 6A, no cytotoxic effect on BMCs was observed at a test concentration up to 100 μg/mL JSOG-6 as determined by the MTT assay (>85% cell survival rate). BMCs were allowed to differentiate into osteoclasts in the presence of RANKL and M-CSF for 5 days. JSOG-6 inhibited the formation of TRAP-positive cells during RANKL-induced osteoclast differentiation in a concentration-dependent manner (Figure 6B). Western blot analysis showed that JSOG-6 also suppressed the protein expression of TRAF6 in RANKL-stimulated cells (Figure 6C).

## Discussion

Osteoporosis is an age-dependent skeletal metabolic disease in which patients suffer from lower bone density and lower bone mass compared to healthy individuals [27,28]. The disease is also associated with a homeostatic imbalance between bone modeling and bone resorption. Although the main cause of the disease is not clear, endocrinologic, nutritional, and genetic factors are thought to be highly involved in osteoporosis.

JSOG-6 is an herbal preparation derived from six traditional medicines that have been clinically used to treat inflammation-associated diseases in oriental Korean medicine. However, the exact pharmacological effect and its underlying mechanisms of action remain to be elucidated. Therefore, the present study was performed to investigate the anti-osteoporotic activity of JSOG-6 in an OVX mouse model and to further elucidate its underlying mechanisms of actions both *in vitro* and *in vivo*.

The OVX animal model has been widely used to study postmenopausal osteoporosis mimicked by estrogen insufficiency [29,30]. Deterioration of trabecular 3D microarchitecture is apparent in the OVX mouse model [31]. We found that JSOG-6 prevented the deterioration of microstructural parameters in the distal femur in mice. Oral administration of JSOG-6 restored bone loss back in OVX mice (Figure 1). These results indicated that JSOG-6 was effective not only in preserving bone mass but also in rescuing the deterioration of bone microarchitecture associated with OVX mice.

Biochemical markers of bone turnover have been determined previously, allowing the evaluation of the status of bone remodeling [32]. Analyses of the serum levels of ALP and OCN, typical biomarkers of osteoblastic activity, and CTx and TRAP, biomarkers of bone resorption, showed that the serum concentrations of ALP, OCN, CTx and TRAP in OVX mice were significantly higher than those in the Sham group. However, the levels of ALP and TRAP in the JSOG-6-treated group were equivalent to the levels in the Sham group (Table 2). These data suggest that JSOG-6 most likely prevents bone loss through decreased bone turnover.

In addition, the mechanisms underlying the cellular effects of JSOG-6 were investigated in osteoblasts and osteoclasts. Osteoblasts, bone-forming cells, synthesize and regulate the deposition and mineralization of the extracellular matrix of bone [33,34]. In this study, JSOG-6 treatment was found to increase the ALP activity in a concentration-dependent manner in MC3T3-E1 cells (Figure 2). JSOG-6 also led to an increase of calcium deposition in MC3T3- E1 cells (Figure 3).

To further explore the mechanism responsible for the effect of JSOG-6 on the regulation of osteoblasts, the markers of bone formation in MC3T3-E1 cells were detected by real-time RT-PCR. LPS leads to the intracellular induction of p38, JNK phosphorylation, and NFκB in macrophages and monocytes, and promotes the differentiation and survival of osteoclasts through the production of several factors such as PGE2, interleukin 1, RANKL, and TNF [35]. The levels of the bone formation biomarkers ALP, OPN, and OPG/RANKL mRNAs were up-regulated by JSOG-6 treatment (Figure 4).

RANKL drives osteoclastogenesis by providing an important signal to osteoclast progenitors through the membrane-anchored receptor RANK in osteoclasts. Osteoblasts also synthesize and secrete OPG, a decoy receptor of RANKL, which blocks the interaction between RANKL and RANK. Therefore, the expression of OPG/RANKL plays an essential role in modulating bone remodeling [36,37]. OPG was also able to block the interaction of RANKL with osteoclast cells, thus suppressing osteoclastogenesis [38]. The data showed that JSOG-6 up-regulated the protein expression of OPG and down-regulated that of RANKL (Figure 5A). Therefore, the suppression of the protein expressions of OPG and RANKL by JSOG-6 might be a plausible partial mechanism of action of its modulation of bone remodeling.

Various intracellular signaling pathways are involved in the regulation of osteoblast differentiation. ERK is considered to be an essential function in the proliferation and differentiation of osteoblasts [39,40]. In this study, JSOG-6 treatment induced the activation of ERK (Figure 5). These findings also suggest that JSOG-6 might have potential in improving bone formation.

Bone formation is regulated by crosstalk between bone-forming osteoblasts and bone-resorbing osteoclasts. Unbalanced osteoclastogenesis causes bone loss in osteoporosis [41,42]. M-CSF and RANKL are important cytokines that cause osteoclast precursors to differentiate into activated osteoclasts [43]. The present study showed that JSOG-6 suppressed RANKL-induced differentiation of osteoclasts by down-regulating the protein expression of TRAF6 in BMCs (Figure 6). These *in vitro* findings were well correlated with the *in vivo* anti-osteoporotic effects of JSOG-6, and thus JSOG-6 might be applicable to the clinic for improving age-dependent bone destruction disease.

## Conclusions

The present study provides evidence that JSOG-6 has a potential anti-osteoporotic activity both *in vitro* and *in vivo*. The underlying mechanisms of action of JSOG-6 are correlated with the induction of ALP activation in osteoblasts, the increase in calcified bone matrix, and the reduction of osteoclast formation. These data provide a pharmacological basis for the use of JSOG-6 as a potential therapeutic strategy for protecting against osteoporotic bone loss in clinic.

## Competing interests

The authors declare that they have no competing interest.

## Authors’ contributions

Conceived and designed the experiments: HJC, LC, and SKL. Performed the experiments HJC and LC. Analyzed the data: HJC, LC, and SKL. Contributed reagents, materials and analysis tools: HJC, LC, JSS, JL, IHH, and SKL. Wrote the paper: HJC, HJP, and SKL. Editing the paper: HJC, HJP, and SKL. All authors read and approved the final manuscript.

## Pre-publication history

The pre-publication history for this paper can be accessed here:

http://www.biomedcentral.com/1472-6882/14/184/prepub
